# Bone microstructural characteristics or positional changes of condyle head affect short-term condyle head resorption after orthognathic surgery

**DOI:** 10.1038/s41598-024-65077-2

**Published:** 2024-06-20

**Authors:** Kazuaki Miyagawa, Chihiro Arikawa, Koichi Hayashi, Soju Seki, Yusuke Yokota, Kazuma Harada, Susumu Tanaka, Emiko Tanaka Isomura

**Affiliations:** 1 Department of Oral and Maxillofacial Surgery, Graduate School of Dentistry, Osaka University, 1-8 Yamadaoka, Suita City, Osaka 565-0871 Japan; 2 Unit of Dentistry, Osaka University Hospital, Suita City, Osaka 565-0871 Japan

**Keywords:** Diseases, Medical research, Risk factors

## Abstract

Condylar resorption occurs in some cases after orthognathic surgery, and the risk factors associated with postoperative condylar head resorption have been extensively described. Nevertheless, even in cases with a combination of risk factors, postoperative condylar resorption may not appear. This study analyzed the microstructure and three-dimensional positional change of the condylar bone via imaging in patients who have undergone bimaxillary orthognathic surgery to determine whether the microstructure or condylar position differs between patients with and without postoperative condylar resorption. Among asymptomatic patients who underwent bimaxillary surgery between April 2021 and March 2022 at our department, 17 patients were analyzed, limited to “female,” “skeletal Class II,” and “high-angle cases,” which are known risk factors for mandibular head resorption. Multidetector computed tomography was performed on these patients before and 6 months after surgery, and the bone microstructure of the condylar head and the three-dimensional positional changes of the condylar bone and the proximal bony fragments were compared with the presence of postoperative condyle resorption using the bone morphology software TRI/3D-BON. Patients with condylar bone abnormalities before surgery and those with high trabecular bone density can develop postoperative resorption if the condyle is misaligned by surgery.

## Introduction

There are some osseous temporomandibular joint (TMJ) diseases with bone resorption such as osteoarthrosis (OA) and progressive condylar resorption (PCR) [also known as idiopathic condylar resorption (ICR)], and some of the cases may be progressive. Although the causes of these diseases are generally unknown, orthognathic surgery has been indicated as one of the causes of resorptive changes in the morphology of the mandibular head due to the excessive burden on the condylar bone caused by the advancement of the mandible. Nevertheless, orthognathic surgery for improving occlusion has also been cited as one of the treatments for PCR, contributing to the confusion among clinicians^1–3^.

The first report of PCR was published by Phillips et al. in 1978, who described a case of bilateral mandibular head resorption after sagittal division of the mandibular branch^1^. Moore et al. also reported the occurrence of retroversion and condylar resorption after anterior mandibular translation, also mentioning the risk factors such as female sex, age of 20–30 s, high preoperative mandibular plane angle, and the presence of preoperative TMJ diseases^2^. Moreover, several studies have described other factors concerning PCR after bilateral sagittal split osteotomy, such as facial morphology, amount of advancement, stretching of the surrounding soft-tissue components producing a posterior facial height, rotation of the proximal segment, duration and method of maxillomandibular fixation, preoperative orthodontic treatment, and the surgeon’s experience^4–9^. There have also been several studies on postoperative condylar resorption for bimaxillary osteotomies, with risk factors comprising female sex, skeletal Class II, open bite, high angle, preoperative condylar in a posterior position in the TMJ fossa, and preoperative abnormal condylar bone morphology^10–17^. Nonetheless, there have been no reports of changes in the bone itself, such as the bone microstructure of preoperative and postoperative condylar bone. Recently, the use of CT has become more common, allowing routine imaging before and after osteotomy to observe the bone in detail. Hence, analysis can be performed concerning the three-dimensional parameters of the trabecular bone or a variety of structural indexes of bony tissue, and some studies have also reported on the bone microstructure after other surgeries^18–21^.

With the increase in the number of bimaxillary osteotomy cases, we have also encountered some cases of postoperative condylar resorption in our clinic. Furthermore, orthodontists have requested us to perform orthognathic surgery on patients with preoperative condylar resorption. However, even in cases with a combination of risk factors, it became obvious that postoperative condylar resorption may or may not be exacerbated or appear. Arnett et al. evaluated the effects of surgically induced condylar position change on the structure of the mandibular condyle in 61 patients who underwent orthognathic surgery tomographically. Results showed that condylar torqueing or posteriorization by orthognathic surgery leads to condylar resorption and late mandibular relapse^22^. However, there are no reports analyzing the preoperative and postoperative three-dimensional positional changes of the condyle and comparing them with postoperative bone resorption according to the preoperative morphological abnormalities of the condyle.

This study aimed to investigate the risk factors of postoperative resorption of the condylar bone in patients undergoing bimaxillary orthognathic surgery by analyzing the microstructure of the condylar bone with images and the three-dimensional positional changes of the condylar bone and the proximal bony fragments. The predictor variables are ‘pre- and post-operative condylar bone microstructure’ and ‘the three-dimensional positional changes of the condylar bone and the proximal bony fragments’ and the outcome variable is ‘postoperative resorption changes of the mandibular head’.

## Results

**Figure 1:**
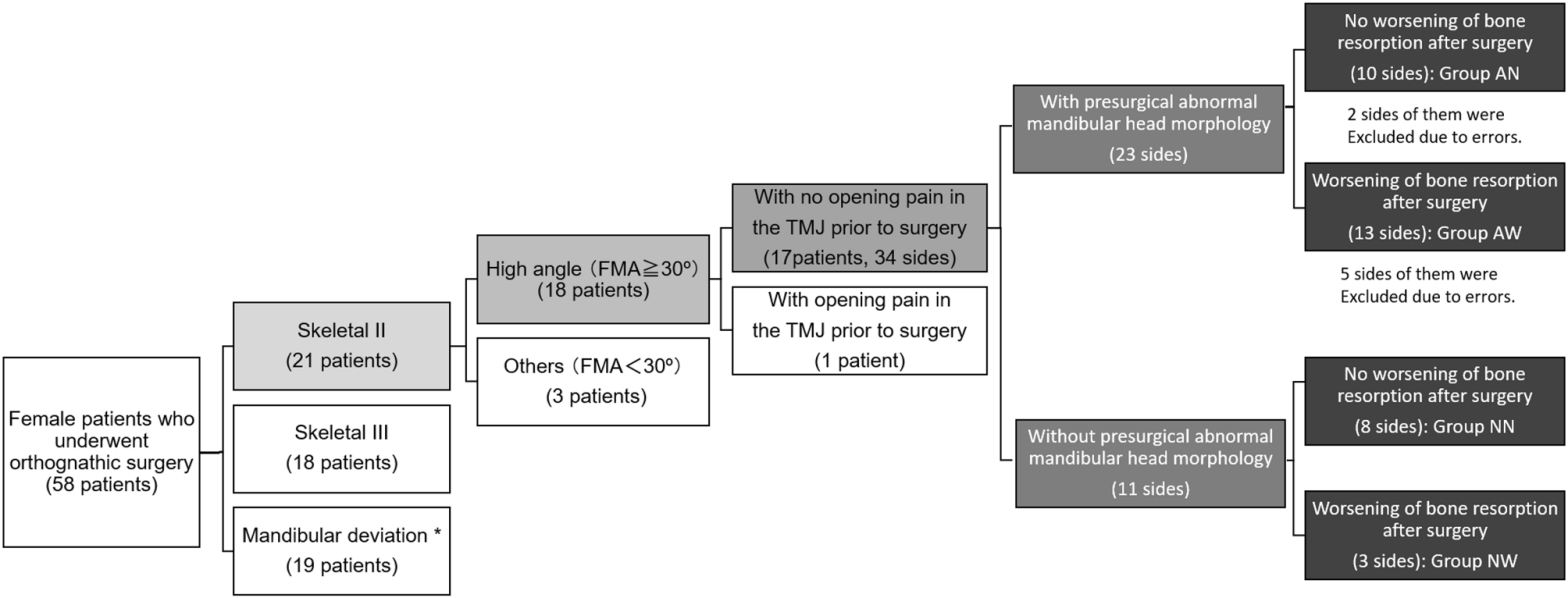
Flowchart of the group of subjects enrolled in this study. Among 90 patients (32 men and 58 women) who underwent orthognathic surgery of the upper and lower jaw in our department between April 2021 and March 2022, 17 women with skeletal Class II high-angle cases (FMA ≧ 30°) and 34 with side mandibular condyle were included. (Some of the patients had preoperative abnormalities on one side and no preoperative abnormalities on the contralateral side and belonged to both groups.). (*Horizontal distance from the midline of the face to the Me is > 4 mm). FMA angle: angle between Frankfurt plane and mandibular submarginal plane.

Among the subjects, 21 of the 58 females were skeletal Class II cases, 18 were skeletal Class III cases, and 19 were mandibular deviation cases (Fig. 1). Among the female skeletal Class II, 18 had a high angle, 17 of whom had TMJ symptoms before surgery. Within that, 15 patients (23 sides) had abnormal mandibular head morphology on multidetector computed tomography (MDCT) before surgery, and 9 patients (11 sides) had normal mandibular head morphology. The group with no postsurgical resorption and presurgical abnormal mandibular head morphology was classified as the “no worsening of bone resorption after surgery (Abnormal morphology-no worsen group: Group AN),” the group with postsurgical resorption and presurgical abnormal mandibular head morphology was classified as the “worsening of bone resorption after surgery (Abnormal morphology-worsen group: Group AW),” the group with no postsurgical resorption and no presurgical abnormal mandibular head morphology was classified as the “no worsening of bone resorption after surgery (Normal morphology-no worsen group: Group NN),” and the group with postsurgical resorption and no presurgical abnormal mandibular head morphology was classified as the “worsening of bone resorption after surgery (Normal morphology-worsen group: Group NW).” Of the 15 patients (23 sides) with presurgical abnormal mandibular head morphology, 13 (8 on the right side and 5 on the left side) exhibited postsurgical mandibular head resorption at 6 months after surgery, accounting for a large proportion of the total resorption group (Group AW). In contrast, Group AN had 10 sides, Group NN had 8 sides, and Group NW had 3 sides. Table 1 shows the background characteristics of the patients. No predominant differences were observed in age, TMJ symptoms, operative time, blood loss, SNA angle [Sella–Nasion–A point (the most concave point of the anterior maxilla) angle], and SNB angle [Sella–Nasion–B point (the most concave point on mandibular symphysis) angle] between the groups. Although 56.5% of patients with preoperative abnormal bone morphology demonstrated worsening resorption after surgery (Group AW), 43.5% showed no changes (Group AN). Furthermore, 27.3% of patients with no preoperative abnormal bone morphology showed postoperative bone resorption (Group NW).

**Table 1:** An overview of the cases analyzed in the present study.

			Group AN	Group AW	Group NN	Group NW	P-value
			(n = 10)	(n = 13)	(n = 8)	(n = 3)	
Age		Average ± SD	32.80 ± 5.11	28.00 ± 6.06	30.62 ± 6.82	31.00 ± 8.54	0.162
		Mean (min, max)	33 (26, 39)	26 (21, 39)	32 (21,39)	32 (22,39)	
TMJ symptoms		Before surgery	0	0	0	0	0.9022
		6 months after surgery	2 (discomfort, pain when opening mouth)	2 (discomfort)	1 (discomfort)	1 (discomfort)	
Operative time (min)		Average ± SD	233.55 ± 42.49	218.56 ± 9.59	218.71 ± 22.09	187.66 ± 39.24	0.544
		Mean (min, max)	215.5 (185, 290)	215 (140, 305)	220.5 (188, 251)	199 (144, 220)	
Blood loss (ml)		Available data	211.66 ± 103.56	211.25 ± 88.65	243.57 ± 145.70	101.66 ± 52.99	0.305
		Mean (min, max)	190 (90, 450)	182 (90, 380)	185 (45, 450)	110 (45, 150)	
SNA angle	Preoperation	Average ± SD	81.60 ± 3.70	83.41 ± 4.88	81.71 ± 3.03	81.66 ± 4.72	0.564
		Mean (min, max)	81 (78, 89)	80 (78, 91)	80 (78, 85)	80 (78, 87)	
	Post operation	Average ± SD	82.00 ± 3.96	83.75 ± 3.86	81.00 ± 2.38	81.66 ± 3.21	0.404
		Mean (min, max)	83 (77, 86)	83 (79, 91)	81 (78, 85)	83 (78, 84)	
	Amount of movement	Average ± SD	0.31 ± 2.00	0.33 ± 2.70	0.28 ± 2.62	0 ± 3.00	0.997
		Mean (min, max)	0 (− 3, 3)	0 (− 4, 4)	0 (− 4, 4)	0 (− 3, 3)	
SNB angle	Preoperation	Average ± SD	73.32 ± 3.23	75.25 ± 4.33	73.57 ± 2.81	73.00 ± 4.35	0.538
		Mean (min, max)	73 (70, 80)	74 (70, 80)	73 (71, 70)	71 (70, 78)	
	Post operation	Average ± SD	75.03 ± 2.50	77.50 ± 4.05	74.85 ± 2.19	76.00 ± 2.64	0.308
		Mean (min, max)	75 (72, 82)	76 (72, 84)	74 (72, 79)	75 (74, 79)	
	Amount of movement	Average ± SD	2.00 ± 0.07	2.25 ± 1.91	1.28 ± 1.38	3.00 ± 2.00	0.395
		Mean (min, max)	2 (1, 3)	2 (−1, 5)	2 (−1, 3)	3 (1, 5)	

SNA angle: Sella–Nasion–A point (the most concave point of the anterior maxilla) angle, SAB angle: Sella–Nasion–B point (the most concave point on mandibular symphysis) angle.

*TMJ* temporomandibular joint.

**Figure 2:**
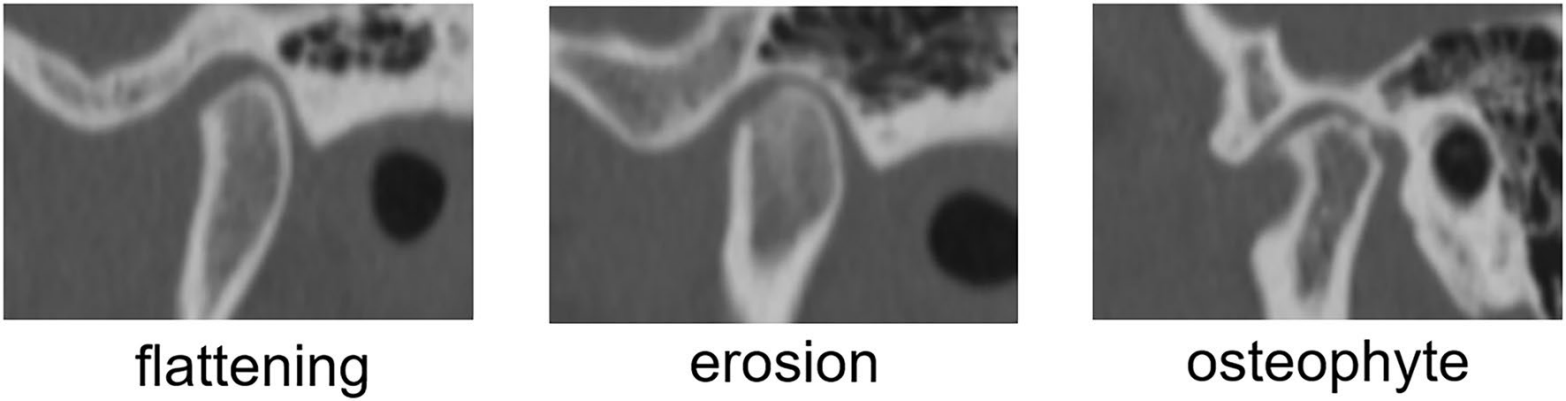
Typical images of “bone resorption”. In the present study, cases of “flattening,” “erosion,” and “osteophyte” bone resorption were recognized. There are other types of bone resorption, but they were not recognized in the present study.

The pattern of the mandibular condylar resorption was articular surface flattening on 8 sides and surface erosion on 2 sides in Group AN. In Group AW, it was articular surface flattening (non-OA) on 12 sides and surface erosion on 1 side preoperatively and surface erosion on 8 sides (all preoperatively articular surface flattening), osteophyte on 2 sides (all preoperatively articular surface flattening), and deviation in form on 1 side (preoperative surface erosion). Some representative images of different examples of “bone resorption” were showed in Fig. 2. Group NW showed articular surface flattening (non-OA) on 1 side and deviation in form on 2 sides. Hence, no difference was found in preoperative bone morphology between Groups AN and AW.

### Analysis of bone microstructure

Two sides in Group AN (both exhibited articular surface flattening before surgery) and five sides in Group AW (two of them exhibited flattening before surgery and osteophyte after surgery, and three of them demonstrated flattening before surgery and erosion after surgery) were excluded because the trabecular bone had no trabecular bone structure due to the loss of trabecular bone beams, causing errors in the TRI/3D-BON analysis.

**Figure 3:**
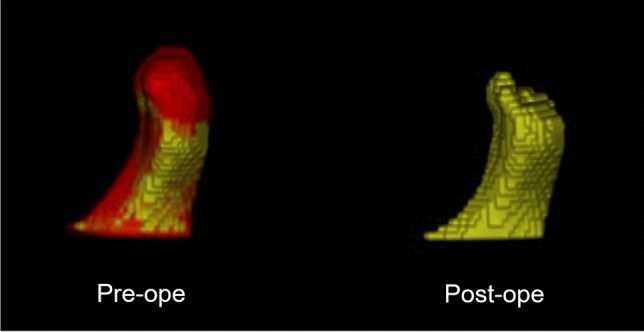
Typical images of the cases of Group AW that could be analyzed with the TRI/3D-BON. The area shown in red in the preoperative image on the left, which was missing when superimposed on the postoperative image, is the area where bone resorption was observed.

Typical images of the cases of Group AW that could be analyzed are shown in Fig. 3.

**Figure 4:**
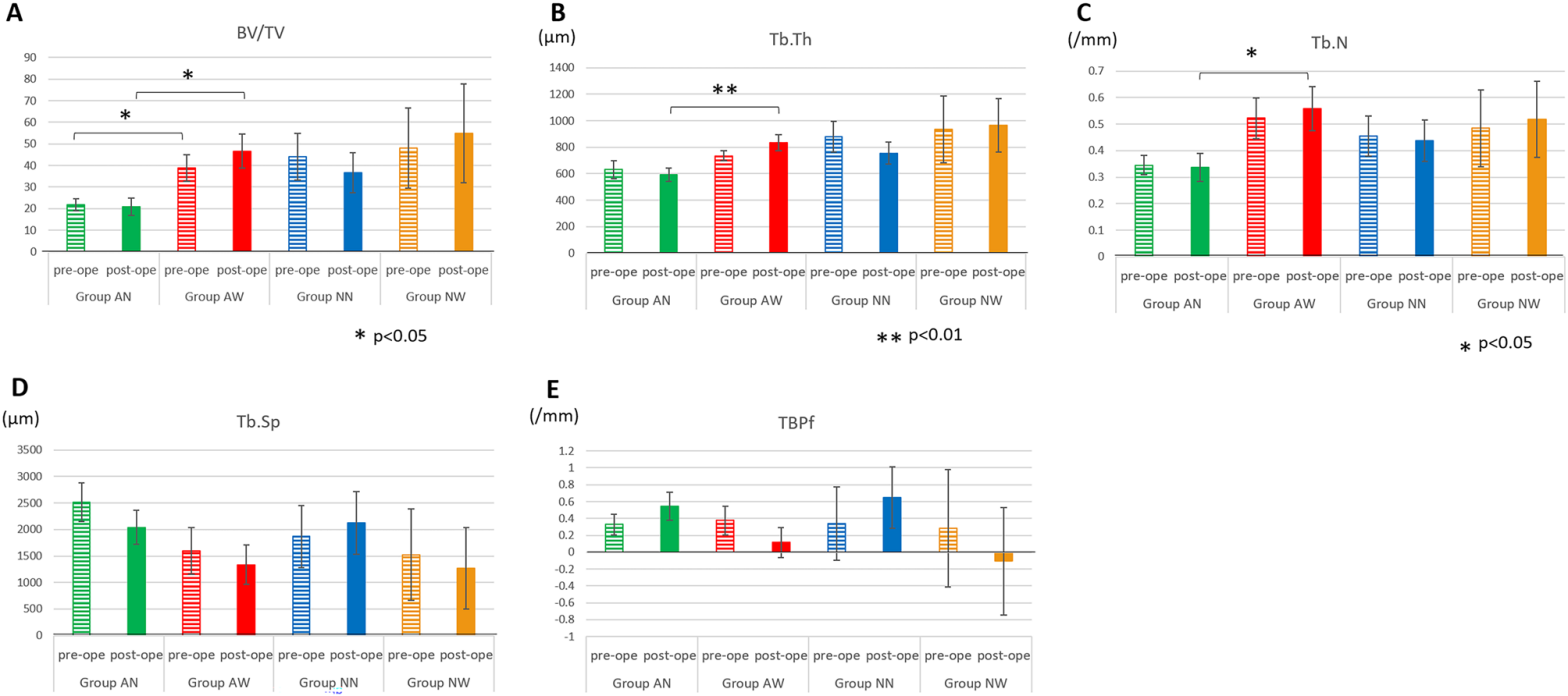
Comparison of bone microstructure among all groups. (**A**) trabecular bone volume density (BT/TV), (**B**) trabecular thickness (Tb,Th), (**C**) trabecular number (Tb.N), (**D**) trabecular separation (Tb.Sp), (**E**) trabecular bone pattern factor (TBPf). In cases with presurgical abnormalities in mandibular head morphology, the mean BV/TV values were 21.79% ± 2.92% and 38.80% ± 6.24% in Groups AN and AW, respectively, before surgery (p < 0.05). After surgery, the mean BV/TV values were 20.80% ± 4.04% and 46.66% ± 7.82% in Groups AN and AW, respectively, (p < 0.05), indicating an increase in postsurgical values compared with presurgical values in Group AW. In both cases with and without presurgical abnormal mandibular morphology, the resorption groups tended to have thicker trabecular width (Tb.Th), greater trabecular number (Tb.N), and narrower trabecular separation (Tb.Sp) than the nonresorption groups. Especially in cases with presurgical abnormal mandibular head morphology, there was a significant difference in the postsurgical Tb.Th with an average of 539.14 ± 51.31 µm in Group AN and 833.923 ± 62.03 µm in Group AW (p < 0.05). A significant difference was also observed in Tb.N with a mean of 0.34 ± 0.05/mm^2^ in Group AN and 0.56 ± 0.08/mm^2^ in Group AW (p < 0.05). These results indicate that Group AW had larger postsurgical Tb.Th, Tb.N, and Tb.Sp than Group AN. Regarding TBPf, among patients with presurgical abnormal mandibular head morphology, the mean TBPf values were 0.33 ± 0.12 and 0.37 ± 0.17 in Groups AN and AW, respectively, before surgery, whereas the respective values after surgery were 0.55 ± 0.17 and 0.11 ± 0.17. Among cases with no presurgical abnormal mandibular head morphology, the mean TBPf values were 0.34 ± 0.44 and 0.28 ± 0.70 in Groups NN and NW, respectively, before surgery, whereas the respective values after surgery were 0.65 ± 0.37 and −0.10 ± 0.63. These results suggest that the mandibular head bone in Group NW, which had a plate-like bone structure before surgery, became denser and exhibited a lattice-like morphology after surgery, whereas that in Groups AN, AW, and NN had a plate-to-rod-like morphology.

In both cases with and without presurgical abnormalities in mandibular head morphology, the trabecular bone volume density (BV/TV) in the mandible of the resorption group was higher than that of the non-resorption group. Especially in cases with presurgical abnormalities in mandibular head morphology, the mean BV/TV values were 21.79% ± 2.92% and 38.80% ± 6.24% in Groups AN and AW, respectively, before surgery (p < 0.05), whereas after surgery, the respective mean BV/TV values were 20.80% ± 4.04% and 46.66% ± 7.82% (p < 0.05), indicating an increase in the mean postsurgical values compared with presurgical values in Group AW (Fig. 4). Further analysis of the bone microstructure in detail show that the resorption groups tended to have thicker trabecular thickness (Tb.Th), greater trabecular number (Tb.N), and narrower trabecular separation (Tb.Sp) than the non-resorption groups in both cases with and without presurgical abnormal mandibular morphology. Especially in cases with presurgical abnormal mandibular head morphology, a significant difference was found in postsurgical Tb. Th with an average of 539.14 ± 51.31 µm in Group AN and 833.923 ± 62.03 µm in Group AW (p < 0.05). A significant difference was also observed in Tb.N with a mean of 0.34 ± 0.05/mm^2^ in Group AN and 0.56 ± 0.08/mm^2^ in Group AW (p < 0.05). These findings indicate that patients in Group AW had larger postsurgical Tb.Th, Tb.N, and Tb.Sp than patients in Group AN.

Regarding the trabecular bone pattern factor (TBPf), among patients with presurgical abnormal mandibular head morphology, the mean TBPf values were 0.33 ± 0.12 and 0.37 ± 0.17 in Groups AN and AW, respectively, before surgery and 0.55 ± 0.17 and 0.11 ± 0.17 in Groups AN and AW, respectively, after surgery. In patients with no presurgical abnormal mandibular head morphology, the mean TBPf values were 0.34 ± 0.44 and 0.28 ± 0.70 in Groups NN and NW, respectively, before surgery and 0.65 ± 0.37 and − 0.10 ± 0.63 in Groups NN and NW, respectively, after surgery. These findings suggest that the mandibular head bone in Group NW, which had a plate-like bone structure before surgery, became denser and exhibited a lattice-like morphology after surgery, whereas that in Groups AN, AW, and NN had a plate-to-rod-like morphology.

### Positional changes of the condylar head

#### Positional change of the condyle in relation to the mandibula fossa

**Figure 5:**
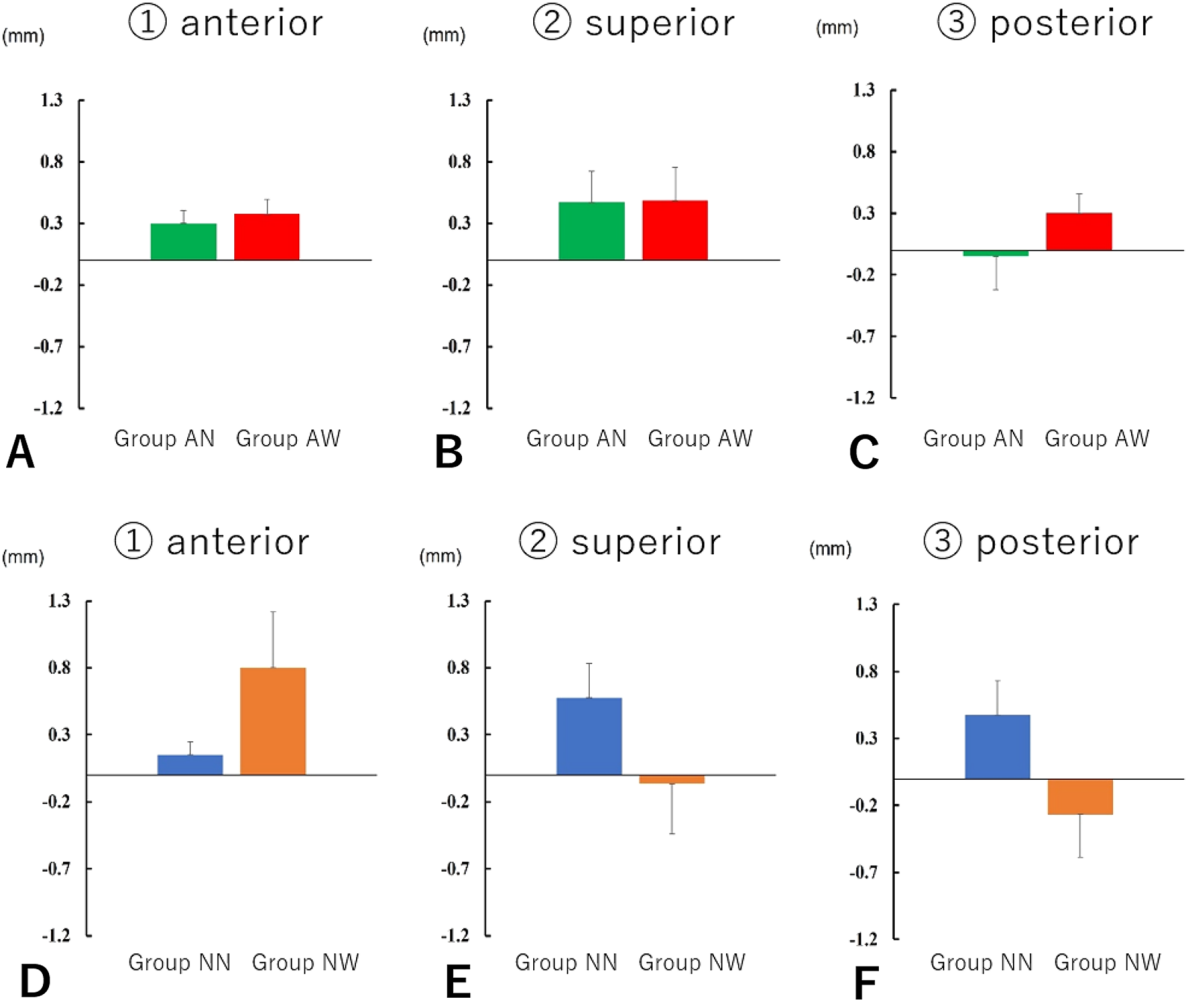
Positional change of the condyle in relation to the mandibula fossa. In patients with preoperative abnormal condylar morphology in Group AN, the postoperative distance between the mandibular fossa and the anterior part of the condyle increased by an average of 0.30 ± 0.10 mm, and the postoperative distance to the superior part increased by an average of 0.47 ± 0.25 mm. The postoperative distance to the posterior part decreased by an average of 0.05 ± 0.27 mm. In patients with preoperative abnormal condylar morphology in Group AW, the anterior distance increased by an average of 0.37 ± 0.11 mm, and the superior distance increased by an average of 0.48 ± 0.27 mm. The posterior distance increased by an average of 0.30 ± 0.15 mm. In patients without preoperative abnormalities in condylar head morphology in Group NN, the distance between the mandibular fossa and the anterior part of the condyle increased by an average of 0.15 ± 0.09 mm, and the distance to the superior part increased by an average of 0.57 ± 0.25 mm. The distance to the posterior part increased by an average of 0.47 ± 0.25 mm. In Group NW, the distance to the anterior part was more likely to increase by an average of 0.80 ± 0.41 mm. The distance to the superior part decreased by an average of 0.06 ± 0.37 mm, and the distance to the posterior part decreased by an average of 0.26 ± 0.31 mm. Results showed that in cases of preoperative abnormal condyle bone morphological abnormalities, the position of the condyle in relation to the mandibular fossa was displaced posteriorly and inferiorly, irrespective of the presence or absence of postoperative bone changes. However, the distance was negligible and not significant. In cases without preoperative condyle morphological abnormalities, the condyle of Group NN was found to be anterior inferior to the mandibular fossa. Meanwhile, the condyle of Group NW was located posteriorly, without significant differences between the two groups.

In patients with preoperative abnormal condylar morphology in Group AN, the postoperative distance between the mandibular fossa and the anterior part of the condyle increased by an average of 0.30 ± 0.10 mm. The postoperative distance to the superior part increased by an average of 0.47 ± 0.25 mm, and the postoperative distance to the posterior part decreased by an average of 0.05 ± 0.27 mm. In patients with preoperative abnormal condylar morphology in Group AW, the anterior distance increased by an average of 0.37 ± 0.11 mm, and the superior distance increased by an average of 0.48 ± 0.27 mm. The posterior distance increased by an average of 0.30 ± 0.15 mm in Group AW. In patients without preoperative abnormalities in condylar head morphology in Group NN, the distance between the mandibular fossa and the anterior part of the condyle increased by an average of 0.15 ± 0.09 mm. The distance to the superior part increased by an average of 0.57 ± 0.25 mm, and the distance to the posterior part increased by an average of 0.47 ± 0.25 mm. In Group NW, the distance to the anterior part increased by an average of 0.80 ± 0.41 mm, and the distance to the superior part decreased by an average of 0.06 ± 0.37 mm. The distance to the posterior part decreased by an average of 0.26 ± 0.31 mm (Fig. 5).

Results showed that in terms of preoperative abnormal condyle bone morphology, the position of the condyle in relation to the mandibular fossa was more likely to be displaced posteriorly and inferiorly, irrespective of postoperative bone changes. However, the distance was negligible and not significant. If there are no preoperative abnormalities in condyle morphology, the condyle of Group NN was found to be anterior inferior to the mandibular fossa. Meanwhile, the condyle of Group NW was located posteriorly. Thus, there was no significant difference between the two groups.

#### Three-dimensional positional changes of the proximal bone segments

##### Change in the sagittal angle of the mandibula ramus

**Figure 6:**
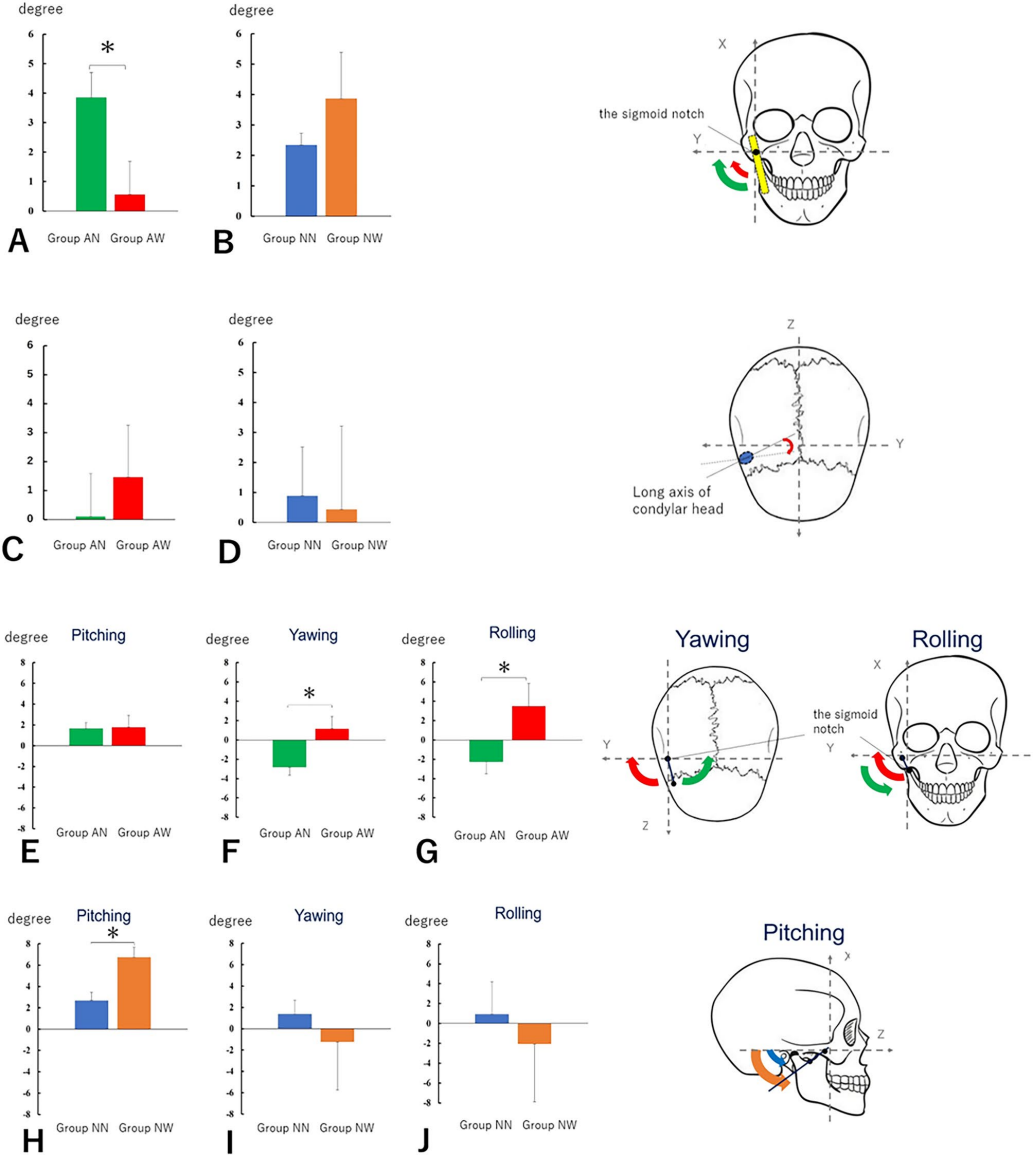
Change in the sagittal angle of the mandibula ramus and the long-axis angle of the condylar head and changes in the three-dimensional position of the coronoid process-mandibular notch. In cases of preoperative abnormal condylar morphology, there was a significant difference (p < 0.05) with a mean sagittal angle of 3.85° ± 0.84° in Group AN and 0.56° ± 1.11° in Group AW. (Fig. 6A, B). Group NN and Group NW did not differ significantly and were similar to Group AN. In patients with preoperative abnormal condylar morphology, Group AN presented with almost no change in the angle. Meanwhile, Group AW had an increase in angle, with an average of 1.46° ± 1.79° (Fig. 6C, D). In patients without preoperative abnormalities in condylar morphology, Group NN had an average angular increase of 0.85° ± 1.63°, and Group NW had an average increase of 0.43° ± 2.78°. In cases involving preoperative abnormalities of the condyle morphology, no significant differences were found in pitching between Group AN and Group AW (Fig. 6E). The average angles during yawning were −2.81° ± 0.82° for Group AN and 1.16° ± 1.28° for Group AW, with the former having a negative rotation and the latter a positive rotation (p < 0.05) (Fig. 6F). In rolling, the average angles were −2.24° ± 1.24° for Group AN and 3.49° ± 2.35° for Group AW, with the former having a negative rotation and the latter having a positive rotation. Hence, the results significantly differed (p < 0.05) (Fig. 6G). Based on these results, Group AN and Group AW presented with inward and outward displacement around the X-axis, respectively. Further, Group AN and Group AW presented with inward and outward displacement around the Z-axis, respectively. In cases without preoperative condylar morphological abnormalities, a significant difference was found in pitching, with an average of 2.68° ± 0.77° in Group NN and 6.73° ± 0.94° in Group NW (p < 0.05) (Fig. 6H). Based on these results, Group NW had a greater counterclockwise rotation of the proximal segment than Group NN. In yawing and rolling, no significant differences were found between Group NN and Group NW (Fig. 6I, J).

In cases of preoperative abnormal condylar morphology, the mean sagittal angle of Group AN was 3.85° ± 0.84°, and the mean sagittal angle of Group AW was 0.56° ± 1.11°. Hence, the results significantly differed (p < 0.05) (Fig. 6A, B). Compared with Group AW, Group AN had a greater lateral displacement of the inferior margin of the mandible, with the mandibular notch as the center of the axis of rotation. Group NN and Group NW did not differ significantly and were similar to Group AN.

##### Change in the long-axis angle of the condylar head

In patients with preoperative abnormal condylar morphology, Group AN presented with almost no change in the angle with an average of 0.10° ± 1.48°. Meanwhile, Group AW presented with increased long-axis angle with an average of 1.46° ± 1.79° (Fig. 6C, D). The average angular increase in patients without preoperative condylar morphological abnormalities in Group NN was 0.85° ± 1.63°. The average angular increase in patients without preoperative condylar morphological abnormalities in Group NW was 0.43° ± 2.78°. However, the results did not significantly differ between the two groups.

##### Changes in the three-dimensional position of the coronoid process-mandibular notch

In cases of preoperative condyle morphological abnormalities, the average angles of Group AN and Group AW during yawning were −2.81° ± 0.82° and 1.16° ± 1.28°, respectively, with former having a negative rotation and the latter a positive rotation (p < 0.05) (Fig. 6F). During rolling, the average angles of Group AN and Group AW were −2.24° ± 1.24° and 3.49° ± 2.35°, respectively, with the former having a negative rotation and the latter a positive rotation. Hence, the results significantly differed (p < 0.05) (Fig. 6G). Based on these results, Group AN and Group AW presented with inward and outward displacement around the X-axis, respectively. Meanwhile, Group AN and Group AW presented with inward and outward displacement around the Z-axis, respectively.

In cases without preoperative condylar morphological abnormalities, a significant difference was found in pitching, with an average of 2.68° ± 0.77° in Group NN and 6.73° ± 0.94° in Group NW (p < 0.05) (Fig. 6H). Based on these results, the proximal segment in Group NW had a greater counterclockwise rotation than that in Group NN. There were no significant differences between Group AN and Group AW for pitching, and Group NN and Group NW for yawing and rolling (Fig. 6E, I, J).

## Discussion

It is difficult to determine whether postoperative mandibular head resorption is natural or pathological. A certain amount of bone resorption is tolerated, but bone resorption (especially for skeletal Class II) is considered to cause a risk of retroversion. In some cases, bone resorption does not occur, and we do not consider it be an absolute postoperative event; it is better not to have it.

We analyzed the structural characteristics of the mandibular head bone and the three-dimensional positional changes of the condyle to investigate the cause of postsurgical mandibular head resorption in patients who had undergone bimaxillary surgery. The analysis was limited to Class II skeletal female patients with high angulation, in whom mandibular head resorption was frequently observed. Although the number of patients was reduced, it was possible to conduct a more detailed analysis. We observed that 56.5% of patients with preoperative abnormal bone morphology demonstrated worsening bone resorption postoperatively, whereas 43.5% of them showed no changes. Moreover, 27.3% of patients with no preoperative abnormal bone morphology showed postoperative bone resorption. To determine the cause of this difference, we examined the bone microstructure.

Chen et al. showed that cone-beam computed tomography scan was performed preoperatively and 3–5 days postoperatively in patients who underwent mandibular advancement to assess the position of the condyle in relation to the mandibular fossa. Further, they revealed that the condyle was displaced posteriorly and inferiorly immediately after surgery if the condyle was resorbed^23^. This study showed no significant difference in the position of the condyle according to the presence or absence of postoperative bony changes. This result differs from that of previous reports, which showed that the postoperative condyle can be caused by edema in the TMJ because of surgery, which may lead to a posterior downward displacement of the condyle position. However, there were no significant differences in terms of surgical duration or volume of blood loss in our study. Thus, there was no difference in the presence or absence of postoperative condyle resorption due to edema.

We observed that the BV/TV of the mandibular head was higher in Group AW than in Group AN preoperatively, and this trend was even higher postoperatively. The trabecular width and number of trabeculae also increased significantly postoperatively, consistent with the increase in BV/TV. MDCT revealed no obvious cortical sclerosis, but the microstructure exhibited preoperative bone sclerosis. This may have been due to the increased load on the TMJ caused by surgery, resulting in increased resorption. In contrast, Groups NN and NW, with no preoperative abnormalities in mandibular head morphology, demonstrated no significant differences in any of the examined parameters. Therefore, these findings suggested that it was difficult to predict postoperative resorption in patients without preoperative bone morphological abnormalities.

On the TRI/3D-BON used in this study, the two-dimensional cross-sectional images are composed of information in the X and Y coordinates, and these are stacked based on the information in the Z coordinate to obtain a three-dimensional image. In this process, the missing information between each cross-section is complemented by the software. When selecting the analysis portion, parameters such as bone trabecular volume fraction cannot be calculated unless a certain amount of bone trabeculae remain, even in pathological conditions where only bone destruction and resorption are observed. In the present study, seven of the cases with preoperative bone morphology abnormalities were excluded due to errors in the analysis because of significant collapse of the trabecular bone and almost complete disappearance of the trabecular bone beams. The inclusion of these cases may have changed the results, but this is a limitation of this analysis method.

**Figure 7:**
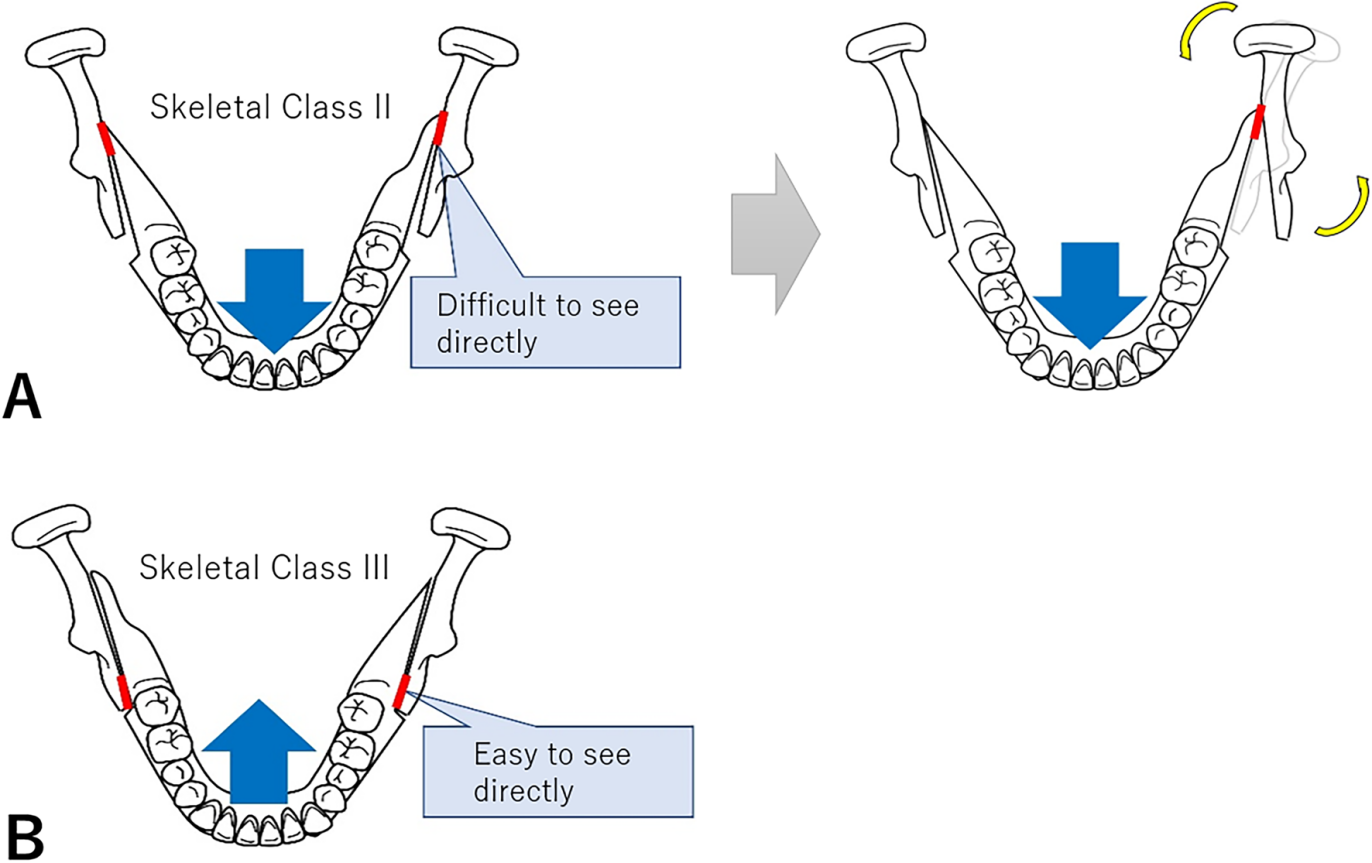
Differences in bone segment interference based on whether the mandible was set forward or back. In skeletal Class II, the interference was more likely to occur at the posterior margin of the distal segment if the distal segment moves forward. If the interference occurs, it rotates around the interference site, displacing the anterior margin of the proximal segment outward and the condyle inward (Fig. 7A). This phenomenon is occasionally difficult to prevent because it is challenging to identify directly during surgery. By contrast, in skeletal Class III, the condylar head is not extremely small originally compared with the mandibular fossa. Moreover, the condylar head is less likely to be displaced. Furthermore, the bony interference is more likely to occur at the anterior margin of the proximal segment, which is easier to detect directly during surgery and less likely to be displaced (Fig. 7B).

Chronic osteomyelitis causes dense hyperostosis of the bone trabeculae and dwarfing of the bone marrow cavity at the site of osteosclerosis^24^. The mandibular head in Group AW similarly exhibited dense hyperostosis of the bone trabeculae and dwarfing of the bone marrow cavity, suggesting that the patient may have poor circulation similar to that observed in the case of chronic osteomyelitis. Moreover, according to several reports, skeletal Class II patients have a smaller mandibular head than skeletal Class III patients, and women have a smaller mandibular head than men^25,26^. Hence, skeletal Class II cases, particularly those with preoperative abnormal bone morphology (resorption), were more likely to have a more an unstable position in the mandibular fossa due to the small size of the condyle in relation to the size of the mandibular fossa. Thus, the condyle is more challenging to position in a centric relation during surgery. Moreover, the position of the condyle within the mandibular fossa is easy to modify, thereby loading the TMJ and potentially causing postoperative resorption of the condyle. In cases of skeletal Class II, the interference was more likely to occur at the posterior margin of the distal segment if the distal segment moves forward. If the interference occurs, it rotates around the interference site, displacing the anterior margin of the proximal segment outwards and the condyle inwards (Fig. 7A). This phenomenon is occasionally challenging to prevent because it is not easy to identify directly during surgery. However, in Group AN, the medial displacement of the condyle was corrected because it was properly guided to the centric relation. Meanwhile, in Group AW, the condyle could not be properly guided to the appropriate position. Hence, condyle resorption may have occurred. By contrast, in skeletal Class III, the condylar head is not extremely small originally compared with the mandibular fossa, and the condylar head is less likely to be displaced. Furthermore, the bony interference was more likely to occur at the anterior margin of the proximal segment, which is easier to identify directly during surgery and is less likely to be displaced (Fig. 7B). Incidentally, at our department, a simplified method for guiding the condyle was used to the centric relation during surgery^27^. After manually guiding the condylar head attached to the proximal segment to posterior and superior to the mandibular fossa, the distance and direction between the marked anterior margin of the mandibular branch and the buccal tube of the maxillary first molar are adjusted similar as before the osteotomy.

To investigate the effect of the deviation and torque of the proximal segment after bony fixation on condyle resorption, the sagittal angle of the mandibular ramus, the long-axis angle of the condylar head, and the three-dimensional positional changes of the proximal segments before and after surgery were analyzed. Results showed that the sagittal angle of the mandibular ramus centered on the mandibular notch in Group AN was significantly greater than that in Group AW. However, this did not lead to postoperative resorption of the condylar head. In the horizontal section of the condylar head, the anterior margin of the proximal fragment was displaced medially in Group AN and laterally in Group AW, centered on the mandibular notch. Meanwhile, in the frontal sections, the inferior margin of the proximal fragment was displaced medially in Group AN and laterally in Group AW around the mandibular notch. Postoperatively, the mandible of Group AW, whose condylar head was worse for resorption, was more likely to be displaced medially. In Group NW, the proximal segments presented with a significant counterclockwise rotation. A counterclockwise rotation of the proximal segment, particularly in the direction of the neck of the condyle backward, is considered a risk factor for condyle resorption^28^. In our study, patients without preoperative abnormalities in condyle morphology had a counterclockwise rotation in Group NW, which fits the risk factors.

Burr et al. reported that different physiological phenomena occur in early and late stages of osteoarthritis, with increased angiogenesis and decreased bone density in the early stage and bone sclerosis due to remodeling imbalance caused by decreased bone resorption without decreased bone formation in the late stage^29^. Group N had a smaller Tb.Th and Tb.N preoperatively and postoperatively. Meanwhile, Group AW had a larger Tb.Th and Tb.N preoperatively and postoperatively. Therefore, patients in Group AN are in the early stage of osteoarthritis, and patients in Group AW are in the late stage.

The mandibular head in Group AW had advanced bone densification, which is a stage of preoperative bone resorption, and may react to even slight postoperative changes in position, causing mandibular condylar resorption due to the load applied to itself. The articular surface is covered with fibrocartilage and underlying subchondral bone, which is sensitive to stress and undergoes extensive remodeling. Excessive mechanical stress is an important factor causing cartilage deterioration and bone resorption in the condylar bone^30^. However, in Group AN, bone densification was less pronounced, indicating that mandibular head resorption is less likely to occur.

Based on these results, as a preventive measure for postoperative condylar resorption, it is predicted that patients with abnormalities in the condylar bone before surgery and those with high trabecular bone density will develop postoperative resorption if the condyle is misaligned by the surgery. Hence, caution should be exercised to avoid loading the condylar bone, and it must be accurately repositioned. Moreover, patients who already had abnormal condylar morphology before surgery might be at a disadvantage concerning the development of condylar resorption after surgery because of the small size and unstable position of the condylar bone with reference to the articular fossa and the increased difficulty in guiding the head to the central position.

Intraoral vertical ramus osteotomy is generally less stressful on the TMJ as the bone segments are not firmly fixed to each other. However, it is not indicated in skeletal Class II as there is no overlap of the bone segments in the forward movement of the mandibular body^15^. One specific measure to guide the condyle to the appropriate position in the mandibular fossa is to select short lingual osteotomy, in which the medial osteotomy in the sagittal division of the mandibular branch is limited to the posterior part of the lingula of the mandible, rather than the osteotomy line extending to the posterior margin of the ramus as in the Dal Pont technique by Obwegeser^31–35^. With this method, the interference site is located anteriorly and is relatively easy to visualize. In addition, if Obwegeser osteotomy is performed, the medial pterygoid muscle is attached to the distal segment, which causes stretching of the medial pterygoid muscle as the segment moves, resulting in retroversion. However, in case of short lingual osteotomy, the medial pterygoid muscle is attached to the proximal segment. Hence, the distal segment is not pulled by the medial pterygoid muscle, thereby preventing the distal bone segment from retroversion. Therefore, short lingual osteotomy is actively used in our department. However, short lingual osteotomy also has some disadvantages. If the emotion of the mucoperiosteal valve, muscles, and tendons of the proximal bone segment is not kept to a minimum, drooping of the condyle, referred to as condylar sag, may occur. Proximal bone segments with less adherent tissues are easily mobile and difficult to guide into a central relation.

To prevent postoperative condylar resorption, MDCT imaging should be performed before the start of orthognathic treatment, and when abnormal condylar morphology or osteosclerosis is already observed, patients should be fully informed of the risk of postoperative condylar resorption in advance. For borderline patients who are eligible for either orthodontic or surgical treatment, it may be necessary to propose nonsurgical treatment options such as camouflage treatment with orthodontic treatment alone. If surgery is selected, caution must be taken to prevent stressing the joints by interfering with bone segments. Medial displacements of the condylar head caused by surgical bone segment interference was also identified as a new risk factor. With the above in mind, it was suggested that even when bone resorption is already present preoperatively, not all patients will develop resorption postoperatively if the osteosclerotic image is not prominent, suggesting that osteotomy is acceptable for patients with PCR, depending on the bone density.

A limitation of this study is the short time period of investigation, which is because only the effects of surgery were considered. Long-term observation requires the consideration of factors other than the effects of surgery, such as the original cause of bone resorption and the effects of postoperative orthodontic treatment. The clinical impression is that condyle head resorption tends to occur in the short term; Jung et al. also reported on condyle head resorption at 6 months and 1 year postoperatively, in which 70% of condyle heads showed resorption at 6 months postoperatively and continued at 1 year^36^. Resorption by volume at 6 months averaged 11 and 17.2% at 1 year postoperatively (6.2% at 7–12 months postoperatively), with significant resorption up to 6 months postoperatively. Furthermore, if images from the 1 year post-operatively are also analyzed in this study, and if the number of resorption cases at 6 months and 1 year post-operatively are different, the grouping will become more complicated and the analysis more complex.

Although MDCT alone was used in this study, MRI may also be effective for evaluating the temporomandibular joint disk and effusion. This is an issue to be considered in the future.

## Materials and methods

The study protocol was approved by the Clinical Ethical Review Committee of Osaka University Dental Hospital, Osaka (Approval Number: R4-E3), and was conducted in compliance with the World Medical Association Declaration of Helsinki on medical research. The same ethics committee (the Clinical Ethical Review Committee of Osaka University Dental Hospital, Osaka) waived informed consent. The participants were allowed to opt out on the hospital website, and patients who opted out of participation were excluded from the analysis.

### Study design and population

This is a retrospective study, and subjects included 90 asymptomatic patients (32 men and 58 women) who underwent bimaxillary orthognathic surgeries (Le Fort I osteotomies and bilateral sagittal split osteotomies) between April 2021 and March 2022 in our department. The subjects were included “female,” “maxillary advancement,” and “high-angle” cases, which are generally considered risk factors for condylar resorption. And they were classified into Class I, Class II, Class III, and mandibular deviation which were asymmetrical jaw cases with a horizontal distance from the midface line to the Menton (Me) of ≥ 4 mm. Among the Class II skeletal patients, an FMA angle (angle between Frankfurt plane and mandibular submarginal plane) of ≥ 30° or more was defined as a high angle, because according to Iwasawa et al., the median FMA angle is 27.28° ± 3.13° for Japanese people with normal occlusion and facial features^37^. And “male,” “mandibular advancement,” “mandibula diviation,” “low-angle” cases, and “case with preoperative TMJ symptoms” were excluded.

MDCT was performed on the study patients before surgery and 6 months after surgery, and the images were analyzed based on 16-bit digital imaging and communication in medicine files. The imaging conditions were 25 × 25 cm (LightSpeed VCT, GE Medical Systems, Japan), and the voxel size was 0.48 × 0.48 × 0.62 mm. During the before-surgery and 1-week postsurgical imaging, the patients were subjected to MDCT with a surgical splint and intermaxillary rubber traction, and during the 6-month postsurgical imaging, the patients were subjected to MDCT without the surgical splint and intermaxillary rubber traction and in the maximal intercuspal position. For each imaging, the bone resorption of the condylar bone before and after surgery was analyzed. Three oral surgeons evaluated the MDCTs, and in cases where the ratings differed, they were reconciled and the ratings were standardised. Bone resorption was present when any of the following signs were found: condylar hypoplasia, condylar hyperplasia, articular surface flattening, subcortical sclerosis, subcortical cyst, surface erosion, osteophyte, generalized sclerosis, loose joint body, deviation in form, and body ankylosis, and the bone was considered normal when none of these signs were found^38^. Among these, “flattening” and “sclerosis” are considered indeterminate signs for diagnosing OA.

The group with no postsurgical resorption with presurgical abnormal mandibular head morphology was classified as the “no worsening of bone resorption after surgery (Group AN),” the group with postsurgical resorption with presurgical abnormal mandibular head morphology was classified as the “worsening of bone resorption after surgery (Group AW),” the group with no postsurgical resorption with no presurgical abnormal mandibular head morphology was classified as the “no worsening of bone resorption after surgery (Group NN),” and the group with postsurgical resorption with no presurgical abnormal mandibular head morphology was classified as the “worsening of bone resorption after surgery (Group NW).” Some of the patients belonged to both groups, i.e., unilateral mandibular head with abnormal presurgical morphology and contralateral mandibular head without abnormal presurgical morphology.

### Analysis of bone microstructure

Bone trabeculae are reticular or linear structures found within trabecular bone and associated with the direction of loading, and their number and thickness are related to bone strength. We analyzed the bone trabecular structure before and after surgery to determine its relationship with postsurgical mandibular head resorption. Thus, female patients with or without preoperative abnormalities in condylar morphology in cases of high-angle maxillary protrusion were compared for bone microstructure with or without worsening of postoperative condylar resorption.

After extracting only the mandibular head up to the height of the mandibular incision using the bone morphology software TRI/3D-BON (RATOC System Engineering, Tokyo, Japan), we compared the following measurements between groups and statistically analyzed the results using Student’s t-test: trabecular bone volume density (BV/TV), trabecular thickness (Tb.Th), trabecular number (Tb.N), and trabecular separation (Tb.Sp). BV/TV is the percentage of trabecular bone volume in tissue volume (trabecular bone volume + bone marrow volume), Tb.Th is the averaged local thickness of trabecular bone, Tb.N is the average number of bone beams per mm unit of trabecular bone, and Tb.Sp is the local thickness of the bone marrow space between trabecular bone^39^.

We also analyzed the trabecular bone pattern factor (TBPf) as an index to evaluate bone beam structure in three dimensions. TBPf is calculated from the ratio of the increase in surface area (ΔS) to the increase in volume (ΔV) when the area of the bone beam (surface area: S) is inflated outward (expansion width: Δr) according to the curvature of the surface (surface curvature: r) and is expressed as follows:

$$ {\text{TBPf }} = \Delta {\text{S}}/\Delta {\text{V}} $$

(ΔS = increase in surface area = postinflated bone surface area − preinflated bone surface area).

(ΔV = increase in volume = postinflated bone volume − preinflated bone volume).

The smaller the value of TBPf, the better the connectivity, and the concave structure has the highest connectivity (TBPf < 0) and becomes plate-like (TBPf ≒ 0), rod-like (TBPf > 0), and spherical (TBPf >  > 0) as the bone beam is destroyed.

(Concave structure: the bone marrow cavity is partitioned into a honeycomb shape by the trabecular bone of the plate. Small chambers are surrounded by concave walls. Plate-like structure: the trabecular bone extends plate-like. The bone marrow cavity is not partitioned into small chambers. Rod-like structure: it extends in the form of a rod. Spherical structure: spherical trabecular bones are connected in a dumpling shape)^40^. Incidentally, this analysis excluded those with significant disintegration of the trabecular bone, where the trabecular bone beams’ structure could not be determined, and measurements were impossible.

### Positional changes of the condylar head

#### Positional change of the condyle in relation to the mandibula fossa

**Figure 8:**
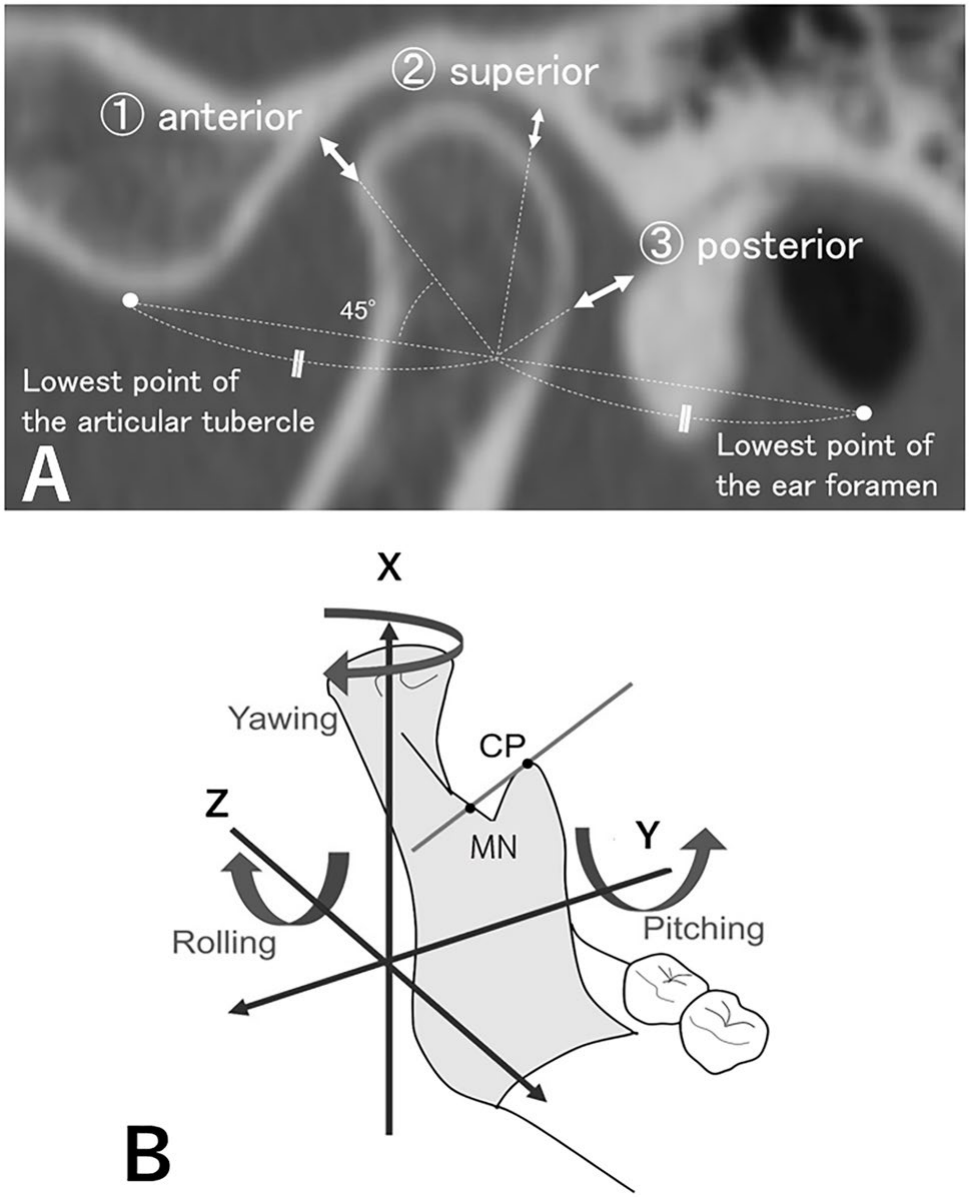
Difference in distance between the mandibular fossa and the condyle and changes in the three-dimensional position of representative line of the mandibular proximal segment. The difference in the distance between the anterior, posterior, and superior mandibular fossa and the condyle was measured at 45° intervals, using the lowest point of the articular tubercle and the midpoint of the tangent line to the lowest point of the ear foramen as reference points in the measured cross-section (Fig. 8A). The changes in the three-dimensional position of the representative line of the mandibular proximal segment were measured preoperatively and postoperatively, based on the study of Jung et al. (Fig. 8B). The representative line of the mandibular proximal segment was set as a reference line through the uppermost point of the coronoid process (CP) and the lowest point of the mandibular notch (MN), and the angles of rotation in the X-axis (Yawing), Z-axis (Pitching), and Z-axis (Rolling) were measured respectively.

The position of the condyle in relation to the mandibula fossa was assessed before and after surgery, and its association with postoperative condyle resorption was investigated. MDCT images taken before and 1 week after surgery using SYNAPSE VINCENT® (Fujifilm Corporation, Tokyo, Japan) were constructed three dimensionally and superimposed on the three reference points of the bilateral frontal incisors and bregma. With reference to the Pullinger’s method of measurement, the difference in terms of distance between the anterior, posterior, and superior mandibular fossa and the condyle was measured at 45° intervals, using the lowest point of the articular tubercle and the midpoint of the tangent line to the lowest point of the ear foramen as reference points in the measured cross-section (Fig. 8A)^36^. Differences in preoperative and postoperative measurements were compared between patients with and without condyle resorption on MDCT at 6 months postoperatively. Statistical analysis was performed using the Student’s *t*-test.

#### Three-dimensional positional changes of the proximal bone segments

To investigate the effect of the deviation and torque of the proximal bone segments, which occurs due to bone segment fixation, on condyle resorption, the three-dimensional positional changes of the proximal bone segments before and after the surgery were analyzed. First, the coordinate system was set up as follows: After three-dimensional construction of MDCT images taken preoperatively and 1 week postoperatively using Dolphin 3D (G.C. Orthology, Tokyo, Japan) and superimposition with reference to the three points of bilateral frontal incisions and bregma, the Frankfurt plane was set as the horizontal plane and the sagittal plane via the cribriform crista galli as the midline plane. The vertical axis was set as the X-axis, the horizontal axis as the Y-axis, and the anterior–posterior axis as the Z-axis. Then, the following areas were evaluated, compared between the resorption and nonresorption groups, and statistically analyzed using the Student’s *t*-test.

##### Change in the sagittal angle of the mandibula ramus

The preoperative and postoperative angular change between the mandibular notch and the inferior margin of the mandible was examined. Measurements were taken in the frontal section via the lowest point of the mandibula notch.

##### Change in the long-axis angle of the condylar head

The amount of change in the long-axis angle of the condyle was evaluated before and after surgery. Clockwise rotation on the right side and anticlockwise rotation on the left side were considered as positive rotation.

##### Changes in the three-dimensional position of representative line of the mandibular proximal segment

The changes in the three-dimensional position of representative line of the mandibular proximal segment were measured preoperatively and postoperatively, based on the study of Jung et al. (Fig. 8B)^36^. The representative line of the mandibular proximal segment was set as a reference line through the uppermost point of the coronoid process and the lowest point of the mandibular notch, and the angles of rotation in the X-axis (Yawing), Z-axis (Pitching), and Z-axis (Rolling) were measured respectively.

## Data Availability

Data is provided within the manuscript.
